# Tyrosine Hydroxylase Inhibitors and Dopamine Receptor Agonists Combination Therapy for Parkinson’s Disease

**DOI:** 10.3390/ijms25094643

**Published:** 2024-04-24

**Authors:** Ling Xiao Yi, Eng King Tan, Zhi Dong Zhou

**Affiliations:** 1 National Neuroscience Institute of Singapore, 11 Jalan Tan Tock Seng, Singapore 308433, Singapore; lingxiao_yi@nni.com.sg; 2 Department of Neurology, Singapore General Hospital, Outram Road, Singapore 169608, Singapore; 3 Signature Research Program in Neuroscience and Behavioral Disorders, Duke-NUS Graduate Medical School Singapore, 8 College Road, Singapore 169857, Singapore

**Keywords:** dopamine, dopamine receptor agonist, neurodegeneration, Parkinson’s disease, therapy, tyrosine hydroxylase inhibitor

## Abstract

There are currently no disease-modifying therapies for Parkinson’s disease (PD), a progressive neurodegenerative disorder associated with dopaminergic neuronal loss. There is increasing evidence that endogenous dopamine (DA) can be a pathological factor in neurodegeneration in PD. Tyrosine hydroxylase (TH) is the key rate-limiting enzyme for DA generation. Drugs that inhibit TH, such as alpha-methyltyrosine (α-MT), have recently been shown to protect against neurodegeneration in various PD models. DA receptor agonists can activate post-synaptic DA receptors to alleviate DA-deficiency-induced PD symptoms. However, DA receptor agonists have no therapeutic effects against neurodegeneration. Thus, a combination therapy with DA receptor agonists plus TH inhibitors may be an attractive therapeutic approach. TH inhibitors can protect and promote the survival of remaining dopaminergic neurons in PD patients’ brains, whereas DA receptor agonists activate post-synaptic DA receptors to alleviate PD symptoms. Additionally, other PD drugs, such as N-acetylcysteine (NAC) and anticholinergic drugs, may be used as adjunctive medications to improve therapeutic effects. This multi-drug cocktail may represent a novel strategy to protect against progressive dopaminergic neurodegeneration and alleviate PD disease progression.

## 1. Parkinson’s Disease (PD)

PD is a neurodegenerative disorder with increasing global incidence. The pathophysiological hallmarks of PD include the selective and progressive degeneration of dopaminergic neurons in the substantia nigra pars compacta (SNpc) and the formation of Lewy bodies in the affected brain areas [1]. Dopaminergic neurons have extensively branched axons in the striatum and require large amounts of energy to transmit nervous signals along these branched axons, which are prone to degeneration [2]. The progressive degeneration of dopaminergic neurons can reduce striatal dopamine (DA) contents, contributing to an imbalance between the direct and indirect circuits in the striatum. This imbalance causes hypercholinergic activity, leading to motor dysfunction involving tremors, postural instability, bradykinesia and muscle rigidity [3,4]. At the advanced stage of PD, affected individuals can experience various non-motor symptoms, including cognitive impairment, sleep disturbance, mental disorder and autonomic nerve dysfunction [5]. Usually, as clinical motor symptoms occur at the onset of PD, almost 60% of striatal dopaminergic neurons are lost in PD patients’ brains [6]. So far, the pathogenesis of PD remains unclear. However, accumulating evidence suggests that DA, the neurotransmitter in dopaminergic neurons, can be an endogenous pathogenic factor that interacts with multiple pathological environmental and genetic factors, contributing to PD onset and development [7,8]. 

## 2. The Pathological Roles of the DA-TH Pathway

### 2.1. Dopamine Toxicity Mechanisms

The synthesis of DA starts by converting the amino acid tyrosine to levodopa (L-DOPA) via tyrosine hydroxylase (TH). Subsequently, L-DOPA is decarboxylated to DA by aromatic amino acid decarboxylase (AADC) [7]. In the resting state, the synthesized DA is absorbed and stored in pre-synaptic vesicles in dopaminergic neurons [9]. Under neuron excitation, DA is released from the pre-synaptic membrane vesicles to synaptic clefts, where it binds and activates post-synaptic DA receptors [10]. However, DA is unstable and can undergo oxidation, generating deleterious oxidative metabolites including reactive oxygen species (ROS), DA quinones (DAQs) and 3,4-dihydroxyphenylacetaldehyde (DOPAL) [8]. Postmortem studies suggest that ROS, produced by DA oxidation, can increase the oxidative stress in PD brains, leading to the oxidative modification of nucleic acids, proteins, lipids and glutathione (GSH) [8]. The highly reactive DAQs and DOPAL can covalently conjugate with lysine, cysteine and tyrosine residues of proteins, which, in turn, leads to misfolding, inactivation and aggregation of the affected proteins [8]. Moreover, DA and its derivatives have been shown to be involved in the toxicity of PD-related neurotoxins, such as iron species, rotenone and MPTP [8,11,12]. The increase in DA content in dopaminergic neurons could enhance rotenone- and MPTP-induced toxicity [13,14], whereas the depletion of DA can significantly attenuate the neuronal apoptosis triggered by rotenone and MPTP [15]. As a co-factor of TH, iron can increase TH expression and promote DA generation [16,17]. Iron species, especially free iron ions, can react with DA to form iron–DA complexes, which subsequently promote DA oxidation to generate toxic DAQs and ROS, contributing to dopaminergic neurodegeneration [12].

Furthermore, DA and its toxic metabolites can interact with PD-related genes, including α-synuclein (*SNCA*), leucine-rich repeat kinase 2 (*LRRK2*), PTEN-induced kinase 1 (*PINK1*), *Parkin*, *DJ-1* and glucocerebrosidase-1 (*GBA1*) in dopaminergic neurodegeneration in PD [18,19,20,21,22]. The α-synuclein (α-syn) protein encoded by the *SNCA* gene, which is the main component of Lewy bodies in PD brains, can form deleterious protein aggregates [23]. DA-derived reactive metabolites, including DAQs and DOPAL, can conjugate with α-syn proteins, promote α-syn protein aggregates and stabilize toxic α-syn oligomers, leading to DA dependent α-syn toxicity in PD [24,25,26,27,28]. DA-derived DAQs can covalently react with the cysteine residues of Parkin protein, decrease the solubility of Parkin protein and impair autophagy, eventually resulting in deleterious protein aggregation and dopaminergic neurodegeneration [29,30,31]. DAQs can conjugate with GCase (encoded by the *GBA1* gene) and inhibit its enzymatic activity, leading to lysosomal dysfunction and α-syn protein accumulation [32]. DAQs can covalently modify cysteine residues (Cys 106) of DJ-1 protein, resulting in DJ-1 protein aggregation and inactivation [33]. The aggregation of DJ-1 protein increased the insolubility of DJ-1 protein which had been identified in PD patients’ brains and implicated in PD pathogenesis [33,34]. Recent findings have shown that the TH-DA pathway is involved in LRRK2 and PINK1 relevant dopaminergic neurodegeneration [35]. LRRK2 and PINK1 function as a balanced serine/threonine–protein kinase pair in PD. LRRK2 up-regulates TH expression and promotes DA generation, which can be enhanced by LRRK2 mutations, whereas PINK1 down-regulates TH expression and inhibits DA synthesis, which can be abolished by PINK1 mutations [35]. Either LRRK2 or PINK1 mutations will disturb the balance of LRRK2–PINK1 kinase pair, enhancing TH and DA levels and promoting dopaminergic neurodegeneration [35].

### 2.2. TH Inhibition-Based Strategies

TH is the key rate-limiting enzyme for DA biosynthesis in dopaminergic neurons [36]. TH can be phosphorylated by protein kinases at Ser31 (Serine31) or Ser40 (Serine40) to enhance TH activity and promote DA synthesis [37]. It has been demonstrated that the over-expression of TH alone can lead to dopaminergic neuron impairment and degeneration in cultured neuron cells and bacterial artificial chromosome transgenic mice models [38,39]. The RNAi knockdown of TH remarkably alleviated the rotenone- and mutant α-syn-induced degeneration of dopaminergic neurons in *Drosophila* PD models [40]. Numerous transcription factors, including paired-like homeodomain transcription factor 3 (Pitx3), nuclear receptor related 1 (Nurr1), cAMP response element-binding protein (CREB), activating transcription factor 2, cAMP responsive element modulator-1 (CREM-1) and neuron-restrictive silencer factor (NRSF), have been reported to regulate TH expression [41,42]. The metastasis-associated protein 1 and heterogeneous nuclear ribonucleoprotein K can bind to the promoter of the TH gene to stimulate TH transcription in neuronal cells [43,44]. The levels of TH can also be regulated by aryl hydrocarbon receptor, histone H3 acetylation and DA transporter [45,46,47]. Additionally, potential LRRK2 inhibitors and PINK1 activators may help maintain LRRK2–PINK1 balance and promote dopaminergic neuron survival [8]. These above-mentioned findings suggest that therapeutic strategies targeting the TH-DA pathway to regulate TH activity can counteract DA toxicity and protect DA neurons. Further studies are necessary to investigate the potential protective effects of TH modulators on PD.

Furthermore, previous studies have shown that alpha-methyltyrosine (α-MT), a competitive and reversible TH inhibitor, can down-regulate DA content in dopaminergic neurons and attenuate the death of dopaminergic neurons induced by environmental and genetic pathogenic factors in cultured neuron cells [39,48,49]. Specifically, it has been demonstrated that the overexpression of α-syn in the presence of DA can induce dopaminergic neuronal injury, whereas the suppression of TH by α-MT can ameliorate the neuronal toxicity induced by α-syn [48]. The application of α-MT has been found to alleviate the degeneration of dopaminergic neurons under *PINK1* mutations [39]. More importantly, our recent studies have highlighted that the continuous administration of low-dose α-MT could prevent *LRRK2* mutation-induced dopaminergic neurodegeneration in transgenic *Drosophila* PD models and extend their lifespan [35]. The TH inhibitory effects of α-MT can be reversible, as α-MT-induced movement side effects in human subjects can be reversed after the termination of drug administration or receiving a high-dose of L-tyrosine [50]. As a Food and Drug Administration (FDA) approved clinical drug, low-dose α-MT administration has been proven to be safe for patients without significant adverse effects after long-term application [50]. The oral administration of α-MT was found to inhibit the synthesis of DA in patients with hypertension-induced pheochromocytoma, Huntington’s disease, dystonia and dyskinesia [50,51,52,53]. The applications of α-MT in different models are summarized in Table 1. Taken together, these findings strongly suggest that TH inhibition can be a potential strategy to protect against dopaminergic neurodegeneration in PD patients.

## 3. Current Therapeutic Strategies

As a neurotransmitter, DfethicA can bind and activate DA receptors on post-synaptic DA receptors for signaling transduction. DA receptors are guanine nucleotide-binding protein (G-protein)-coupled receptors that are widely distributed in various regions of the human brain [59]. DA receptors can be divided into two categories: D1-like receptors and D2-like receptors. Both D1-like receptors and D2-like receptors are selectively expressed in striatal medium spiny neurons, with D1 receptors projecting to the globus pallidus interna (direct pathway) and D2 receptors projecting to the globus pallidus pars externa (indirect pathway) [60]. It is generally accepted that the activation of D1-like receptors stimulates adenylyl cyclase (AC) activity, promotes cyclic AMP (cAMP) formation and activates the direct pathway, whereas the activation of D2-like receptors reduces AC activity, suppresses cAMP formation and inhibits the indirect pathway [61,62].

**Figure 1:**
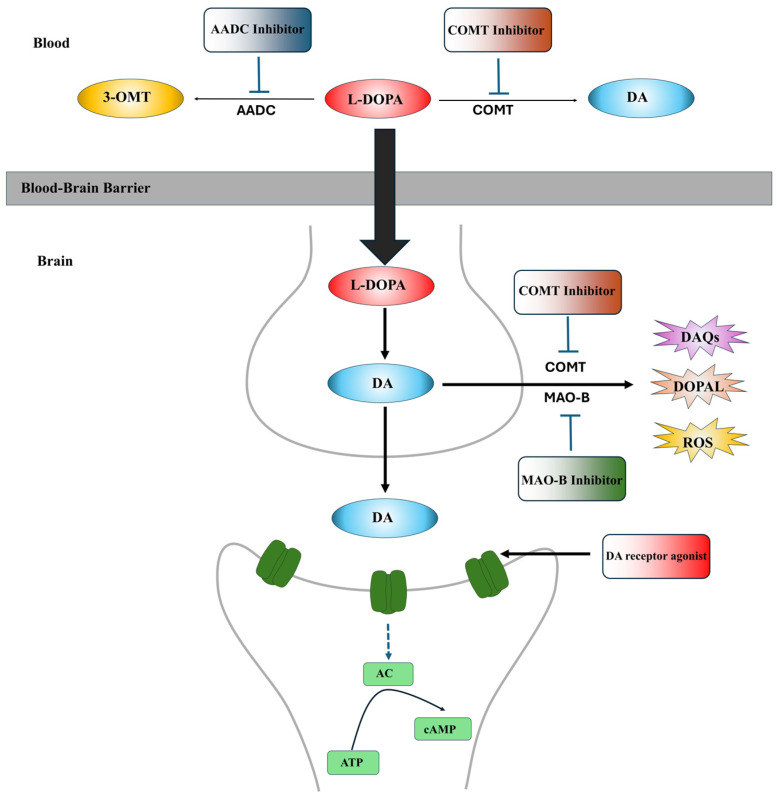
The sites of action of anti-PD medications. L-DOPA is the precursor of DA, which can cross the BBB and enter dopaminergic neurons to increase DA contents in PD brains. AADC inhibitors, such as carbidopa and benserazide, and peripheral catechol-O-methyltransferase (COMT) inhibitors, such as tolcapone and entacapone, are used as adjunct medications to L-DOPA therapy to prevent L-DOPA degradation in the periphery, increase the amounts of L-DOPA to enter dopaminergic neurons and enhance the bioavailability of L-DOPA. In the brain, type-B monoamine oxidase (MAO-B) inhibitors, including selegiline and rasagiline, and central COMT inhibitors, including tolcapone, are commonly applied as adjuvants to L-DOPA therapy to inactivate the MAO and COMT in the synaptic cleft, preventing DA degradation. DA receptor agonists, a class of chemical compounds that function as DA substitutes, directly bind and activate post-synaptic DA receptors to improve the motor and non-motor symptoms of PD.

Currently, the L-DOPA replenishing strategy is the gold standard for clinical PD treatment to alleviate PD symptoms. L-DOPA is the precursor of DA, which can cross the blood–brain barrier (BBB) and enter dopaminergic neurons to increase DA in PD brains [63,64]. The clinical application of L-DOPA can alleviate motor symptoms (tremors, stiffness and bradykinesia) in both early and advanced stages of PD patients [65]. However, long-term treatment with L-DOPA is associated with motor complications, including dyskinesia and motor fluctuations, due to its short plasma half-life [63,66]. In clinical usage, L-DOPA is often administered in combination with peripheral AADC inhibitors, such as carbidopa and benserazide, and peripheral catechol-O-methyltransferase (COMT) inhibitors, such as tolcapone and entacapone, to prevent the conversion of L-DOPA to DA or 3-methoxytyramine (3-OMT) in the periphery, thereby increasing the amount of L-DOPA available to enter dopaminergic neurons and enhancing the bioavailability of L-DOPA [67,68,69]. Type-B monoamine oxidase (MAO-B) inhibitors, including selegiline and rasagiline, and COMT inhibitors, including tolcapone, are commonly used as adjuvants to L-DOPA to inactivate the MAO and COMT in the synaptic cleft, preventing DA degradation. However, these medications cannot completely resolve L-DOPA-related motor complications [70]. Moreover, L-DOPA can be toxic to dopaminergic neurons [71,72]. The oxidation of L-DOPA can generate ROS and DAQs [71,72]. Furthermore, long-term replenishment of L-DOPA to maintain higher DA levels in dopaminergic neurons to alleviate PD symptoms may accelerate DA neurodegeneration and disease progression in PD patients [73,74]. The sites of action of these medications are summarized in Figure 1.

Moreover, DA receptor agonists are alternative drugs to L-DOPA therapy to alleviate PD symptoms. DA receptor agonists are a class of chemical compounds that function as DA substitutes to directly bind and activate post-synaptic DA receptors to improve the motor and non-motor symptoms of PD patients (Figure 1) [75]. Currently, there are ten types of DA receptor agonists approved by various countries for PD treatment, which can be categorized into two groups based on their chemical structure: ergoline agonists (bromocriptine, lisuride, α-dihydroergocryptine, pergolide and cabergoline) and non-ergoline agonists (piribedil, rotigotine, pramipexole, ropinirole and apomorphine) [59]. In addition to the approved DA receptor agonists, several DA receptor agonists targeting D1-like receptors, including PF-06412562, PF-06649751 and PF-06669571, are undergoing clinical trials for PD [76,77,78].

Among the ergoline agonists, bromocriptine, lisuride and α-dihydroergocryptine primarily activate D2-like receptors, whereas pergolide and cabergoline have higher affinities for D2-like receptors and lower affinities for D1-like receptors [79,80]. These ergoline agonists can be used as monotherapy to improve motor symptoms in early PD patients or used as an adjunct drug to L-DOPA treatment in advanced PD patients [81,82,83]. However, these ergoline agonists are no longer recommended for clinical use due to their serious side effects, including mental changes, dyskinesias, peripheral edema, excessive daytime sleepiness, hallucinations, pulmonary fibrosis, valvular heart disease, pleural effusion and pericardial effusion [80,84,85].

Non-ergoline agonists are commonly used for the treatment of PD due to their better safety profile with respect to cardiovascular complications [80]. Piribedil, a selective DA agonist with a higher affinity for D2-like receptors, has been used as monotherapy or as an adjunct drug to L-DOPA therapy in early PD patients without motor fluctuations [86,87,88]. Rotigotine (stimulates both D1-like and D2-like receptors), pramipexole (primarily acts on D2-like receptors) and ropinirole (primarily acts on D2-like receptors) can be used as monotherapy or as adjunct drugs to L-DOPA therapy in early and advanced PD patients [89,90,91,92,93,94,95]. Apomorphine, which has a higher affinity for D2-like receptors and a lower affinity for D1-like receptors, has been recommended as a rescue treatment in advanced PD patients who suffer from drug-resistant OFF time and are not fully controlled by standard oral treatments [96,97]. Common documented adverse effects of these non-ergoline agonists include nausea, yawning, headache, somnolence, dizziness, orthostatic hypotension, application site reactions and daytime sleepiness [80,86,89,92]. Detailed information on the structure, specificity, interaction and side effects of each non-ergoline DA receptor agonist is summarized in Table 2.

## 4. Combination Therapeutic Strategies Based on TH Inhibitors

**Figure 2:**
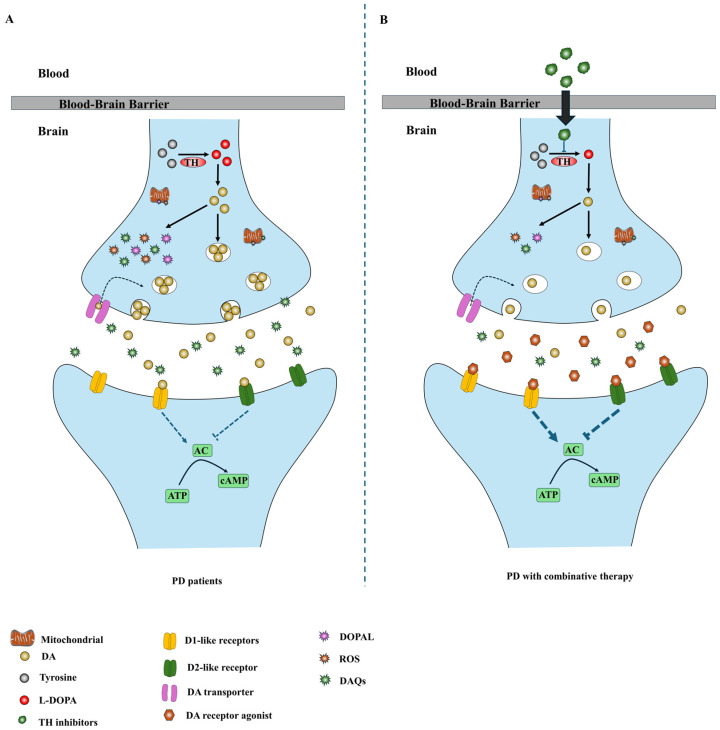
Diagram of combination therapeutic strategies based on TH inhibition. (**A**) In PD patients, the degeneration of dopaminergic neurons in the SNpc reduces the DA in the striatum, resulting in the development of PD symptoms. DA is unstable and can be oxidized to generate multiple deleterious metabolites. These toxic oxidative byproducts can contribute to DA neurodegeneration in PD. (**B**) DA receptor agonists activate post-synaptic DA receptors to alleviate PD symptoms, whereas TH inhibitors suppress DA generation and down-regulate DA levels in the remaining dopaminergic neurons to promote the survival of dopaminergic neurons in PD brains.

So far, no drugs and therapies can alleviate the progressive degeneration of dopaminergic neurons in PD. Here, we propose a combination therapy based on TH inhibitors plus DA receptor agonists for PD. In this combination strategy, DA receptor agonists and TH inhibitors will be conjunctively administered to PD patients. DA receptor agonists activate DA receptors and alleviate PD symptoms, whereas the reversible TH inhibitor α-MT inhibits DA generation, protects the remaining dopaminergic neurons in PD brains and alleviates disease progression. DA receptor agonists have been used as effective drugs to control motor and non-motor symptoms in PD patients. They are expected to be the first-line agents for symptomatic alleviation in early and advanced PD, as well as for delaying or reducing L-DOPA-caused motor complications. The reversible TH inhibitor α-MT has been shown to suppress DA synthesis in multiple studies. Furthermore, α-MT is a clinically approved drug, and it is safe enough for long-term usage in humans. Our combination therapy with DA receptor agonists and the TH inhibitor α-MT may become the first promising strategy to protect against dopaminergic neurodegeneration and delay the progression of PD (Figure 2).

Previous studies have demonstrated that α-MT administration or TH gene inactivation can enhance the locomotor therapeutic effects of DA receptor agonists in mice PD models, suggesting the feasibility of our proposed combination therapy with α-MT and DA receptor agonists for PD [108,109]. The combination therapy may have the most significant therapeutic effects in early-stage PD patients. In the early stages of PD, more dopaminergic neurons remain in patients’ brains compared to advanced-stage PD patients, which can be protected and functioned by the combination therapy. At the advanced PD stage, the remaining dopaminergic neurons, the available dopaminergic neuron synapses and post-synaptic DA receptors will be much less, which may lead to limited therapeutic effects under combination therapy. The combination therapy may also be applicable to inherited PD cases with LRRK2, PINK1 and α-syn gene mutations, as PD gene mutations can disturb the TH-DA pathway and up-regulate DA production, leading to DA-dependent neurodegeneration.

## 5. Limitations and Future Directions

The therapeutic effects of the combination strategy with DA receptor agonists and α-MT need future clinical assessments and investigations. Several hurdles or issues, such as potential drug–drug interactions, the balance between the direct and indirect circuits in the striatum, as well as the cellular redox, need to be addressed in future clinical investigations. α-MT is commonly used to decrease blood pressure in patients with essential hypertension and phaeochromocytoma, with orthostatic hypotension being an infrequent adverse effect [110,111]. Furthermore, acute orthostatic hypotension is one of the common side effects in PD patients receiving DA receptor agonists, especially piribedil, pramipexole and ropinirole [104]. DA receptor agonists can significantly suppress blood pressure, causing a dramatic drop in blood pressure even after the first several doses [104]. Therefore, therapies with α-MT plus DA receptor agonists may aggravate the hypotensive situation in PD patients. Close screening and monitoring of blood pressure should be instituted as a routine precautionary practice during combination therapy for PD patients. The doses of α-MT or DA receptor agonists may need to be adjusted or discontinued according to patients’ responses to drug treatments.

Previous pharmacological investigations had found that DA receptor agonists could significantly elevate DA levels in the brains of healthy rats [112]. Moreover, four DA receptor agonists, including apomorphine, pramipexole, piribedil and bromocriptine, could dose-dependently antagonize α-MT-induced DA level decline in the brains of healthy mice and rats [113,114]. Three clinically approved DA receptor agonists, apomorphine, piribedil and bromocriptine, could dose-dependently reverse low-dose α-MT-induced DA level decline in mice brains [113]. Notably, piribedil at 8 mg/kg and apomorphine at 2 mg/kg nearly abolished the low-dose α-MT-induced reduction in DA level [113]. Pramipexole was found to dose-dependently antagonize α-MT-induced DA decline in the rat striatum [114]. These findings suggest that α-MT-induced DA level decline may be antagonized by DA receptor agonists in the combination therapy, although we do not know how DA receptor agonists antagonize α-MT-induced DA level reduction in animal models. Due to the above-mentioned situations, close and continuous monitoring of DA levels in PD patients’ brains before and after combination drug administration should be indispensable for PD patients undergoing combination therapy. The doses of α-MT and DA receptor agonists need to be adjusted based on changes in DA levels in PD patients’ brains.

It is well known that aging is an important risk factor for PD, accompanied by elevated oxidative stress and suppressed GSH levels in the brain [115]. GSH is the most abundant nonprotein peptide in the body and is responsible for maintaining cellular redox status. The application of GSH as a therapeutic agent is limited by its very short half-life in human plasma and its difficulty in crossing cell membranes [116]. Under physiological conditions, the cellular availability of cysteine is considered to be the rate-limiting factor in the synthesis of intracellular GSH [117]. N-acetylcysteine (NAC) is an FDA-approved antioxidative medication, which can be systemically administered to increase cysteine levels in the brain and promote the synthesis of GSH [117]. Administration of NAC has been shown to be a safe and effective adjunct therapy in patients with psychiatric disorders who received antipsychotic medications, such as risperidone, chlorpromazine and trihexyphenidyl [118,119]. The neuroprotective effects of NAC in PD have been highlighted in preclinical and clinical studies. Long-term administration of NAC substantially reduced neuronal loss, oxidative stress and motor abnormalities in PD mouse models [120,121]. Administering a single dose of NAC (150 mg/kg) in PD subjects has been found to increase GSH levels in the blood and brain [122]. Recently, clinical studies have indicated that weekly intravenous infusions of NAC (50 mg/kg) plus oral administration (500 mg twice per day) for three months can improve the clinical symptoms of PD and increase the binding of DA to transporters in the caudate and putamen of PD patients [123,124]. These findings indicate that NAC therapy may positively affect the dopaminergic system in PD patients, which could be used as an adjunctive therapy to enhance the clinical efficacy of the combination therapy in PD.

DA and acetylcholine (ACh) are two major neurotransmitters in the basal ganglia circuits, which play a vital role in regulating motor symptoms [125]. In PD, the balance between DA-ACh is disrupted due to the decrease in DA levels in the striatum, resulting in hypercholinergic activity and motor and non-motor symptoms [4]. Meanwhile, anticholinergic drugs, inhibiting the ACh pathway in the brain, have long been used to manage motor symptoms in PD before the development of L-DOPA therapy, and these drugs are still used clinically for PD [126]. Anticholinergic drugs have adverse side effects, including blurry vision, dry mouth, urinary retention, confusion, cognitive and memory problems, restlessness and hallucinations and stimulation of locomotor activities [127,128]. However, locomotor behavior stimulated by anticholinergic drugs can be inhibited by the oral administration of α-MT [129]. It is proposed that anticholinergic drugs may act as adjunctive medications to enhance therapeutic effects. Further investigations are necessary to evaluate the beneficial effects of anticholinergic drugs in combination therapy.

## 6. Conclusions

TH inhibitors down-regulate DA levels and protect DA neurons, while DA receptor agonists function to activate post-synaptic DA receptors to alleviate PD symptoms. NAC and anticholinergic drugs may be added to improve therapeutic effects. A multi-drug combination therapy including TH inhibitors should be further explored in clinical trials in PD patients.

## Figures and Tables

**Table 1:** The applications of α-MT in disease models.

Disease	Models	Clinical Outcomes	Reference
PD	Dopaminergic cell models	Ameliorates overexpression of α-syn induced neurotoxicity	[48]
PD	Dopaminergic cell models	Ameliorates *SNCA* mutant-induced neurotoxicity	[54]
PD	Dopaminergic cell models	Ameliorates *PINK1* mutant-induced neurotoxicity	[39]
PD	Dopaminergic cell models	Ameliorates proteasome inhibitor-induced neurotoxicity	[49]
PD	Transgenic *Drosophila* model	Ameliorate *LRRK2* mutant-induced neurodegeneration and extent of lifespan	[35]
Dystonia	Human patients	Well-tolerated and attenuates hallucinations and painful dystonia	[50]
Dystonia	Human patients	Well-tolerated and improves physical signs of tardive dystonia	[50]
Dyskinesia	Human patients	Well-tolerated and improves physical signs of tardive dyskinesia	[50,55]
Pheochromocytoma	Human patients	Well-tolerated and relieves symptoms of Pheochromocytoma	[51]
Huntington’s disease	Human patients	Improves movement symptoms	[56]
Schizophrenics	Human patients	Well-tolerated and potentiates the therapeutic effects of antipsychotic medications	[57]
Infantile Spasms	Human patients	Relieves physical symptoms	[58]

**Table 2:** The structure, interactions, clinical use and side effects of the non-ergoline DA-receptor agonists.

Name	Formula	Structure	Specificity	Clinical Use	Side Effects	References
Piribedil	C_16_H_18_N_4_O_2_	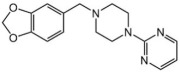	D2-like receptor	Monotherapy or adjunct drug to L-DOPA therapy in early PD patients without motor fluctuations	Nausea, vomiting, confusion, agitation, dizziness, hypotension, orthostatic	[86,87,88,98]
Rotigotine	C_19_H_25_NOS	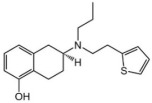	D1-like and D2-like receptors	Monotherapy or adjunct drugs to L-DOPA therapy in both early and advanced PD patients	Nausea, application site reactions, dizziness, insomnia, somnolence, vomiting, fatigue and orthostatic hypotension	[89,90,99,100,101]
Pramipexole	C_10_H_17_N_3_S	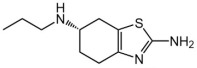	D2-like receptors	Monotherapy or adjunct drugs to L-DOPA therapy in both early and advanced PD patients	Sleep attack, nausea, somnolence, fatigue and orthostatic hypotension	[91,102,103,104]
Ropinirole	C_16_H_24_N_2_O	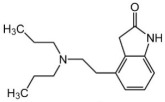	D2-like receptors	Monotherapy or adjunct drugs to L-DOPA therapy in both early and advanced PD patients	Orthostatic hypotension, dizziness, nausea, somnolence, sleep attacks	[92,93,94,95,105]
Apomorphine	C_17_H_17_NO_2_	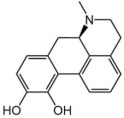	D1-like and D2-like receptors	Advanced PD patients who suffer from drug-resistant OFF time	Yawning, headache, drowsiness, nausea, dizziness, postural instability, injection site reactions	[105,106,107]

## Data Availability

No research data available in this manuscript.
